# Population Pharmacokinetic Study of Cefathiamidine in Infants With Augmented Renal Clearance

**DOI:** 10.3389/fphar.2021.630047

**Published:** 2021-03-15

**Authors:** Bin Du, Yue Zhou, Bo-Hao Tang, Yue-E Wu, Xin-Mei Yang, Hai-Yan Shi, Bu-Fan Yao, Guo-Xiang Hao, Dian-Ping You, John van den Anker, Yi Zheng, Wei Zhao

**Affiliations:** ^1^ Department of Clinical Pharmacy, Key Laboratory of Chemical Biology (Ministry of Education), School of Pharmaceutical Sciences, Cheeloo College of Medicine, Shandong University, Jinan, China; ^2^ Department of Clinical Pharmacy, Clinical Trial Center, The First Affiliated Hospital of Shandong First Medical University and Shandong Provincial Qianfoshan Hospital, Jinan, China; ^3^ Pediatric Research Institute, Children’s Hospital of Hebei Province Affiliated to Hebei Medical University, Shijiazhuang, China; ^4^ Division of Clinical Pharmacology, Children’s National Medical Center, Washington, DC, United States; ^5^ Departments of Pediatrics, Pharmacology & Physiology, Genomics and Precision Medicine, The George Washington University School of Medicine and Health Sciences, Washington, DC, United States; ^6^ Department of Paediatric Pharmacology and Pharmacometrics, University Children's Hospital, University of Basel, Basel, Switzerland

**Keywords:** cefathiamidine, pharmacokinetics, dosing, infants, augmented renal clearance

## Abstract

**Objectives:** Augmented renal clearance (ARC) of primarily renally eliminated antibacterial agents may result in subtherapeutic antibiotic concentrations and, as a consequence, worse clinical outcomes. Cefathiamidine is frequently used as empirical antimicrobial therapy in children with ARC, but pharmacokinetic studies in infants are lacking. This population pharmacokinetic study in infants with ARC was conducted to determine optimal dosing regimens of cefathiamidine.

**Methods:** The population pharmacokinetics was conducted in 20 infants treated with cefathiamidine. Plasma samples of cefathiamidine were collected using opportunistic sampling, and the concentrations were detected by UPLC-MS/MS. Data analysis was performed to determine pharmacokinetic parameters and to characterize pharmacokinetic variability of cefathiamidine using nonlinear mixed effects modelling (NONMEM) software program.

**Results:** The data (*n* = 36) from 20 infants (age range, 0.35–1.86 years) with ARC were fitted best with a 1-compartment model. Allometrically scaled weight and age as significant covariates influenced cefathiamidine pharmacokinetics. The median (range) values of estimated clearance and the volume of distribution were 0.22 (0.09–0.29) L/h/kg and 0.34 (0.24–0.41) L/kg, respectively. Monte Carlo simulations showed that the cefathiamidine doses of 100 mg/kg/day q12 h, 50 mg/kg/day q8 h and 75 mg/kg/day q6 h were chosen for bacteria with MIC 0.25, 0.5 and 2 mg/L, respectively.

**Conclusion:** The population pharmacokinetic model of cefathiamidine for infants with ARC was developed. The PTA - based dosing regimens were recommended based on the final model.

## Introduction

Cefathiamidine is a first-generation cephalosporin discovered in 1974 and is used to treat infections in pediatric patients (National pediatric multi-center cooperative group of cefathiamidine observation, 2003). According to epidemiological studies, cefathiamidine is one of the most commonly prescribed antimicrobial drugs in Chinese pediatric hospitals (Zhang et al., 2008a; Zhang et al., 2008b; Fan et al., 2019). The database from the China Medical Information Center showed that cefathiamidine was the fourth most frequently used cephalosporin in 2016. It has broad antibacterial activity against *Enterococcus*, *Streptococcus pneumoniae*, *Branhamella catarhalis*, *Streptococcus pyogenes, Methicillin-sensitive Staphylococcus epidermidis* (MSSE), *Haemophilus influenza, and Methicillin-sensitive Staphylococcus aureus* (MSSA) (Tze-ying et al., 1979; Chen and Williams, 1983). It has a protein binding capacity of 23% and is excreted primarily in unchanged form through the renal route (> 90%) within 12 h after intravenous administration (Tze-ying et al., 1979). Hence, the kidney function is a crucial factor affecting the pharmacokinetics of cefathiamidine.

Augmented renal clearance (ARC) is a phenomenon in critically ill adult and pediatric patients characterized by increased creatinine clearance and elimination of renally eliminated drugs (Heggen et al., 2019). However, there is no uniform ARC criterion for pediatric patients. ARC was defined based on estimated glomerular filtration rate (eGFR) ≥ 130 ml/min/1.73 m^2^ in pediatric patients (Béranger et al., 2018; Béranger et al., 2019; Lv et al., 2020). ARC is strongly associated with subtherapeutic concentrations of antibiotics such as β-lactams and vancomycin, which leads to underexposure and, as a consequence, to increased treatment failure (Udy et al., 2012; Carlier et al., 2013; Udy et al., 2015; Lv et al., 2020). ARC is likely to influence the pharmacokinetic (PK) parameters of cefathiamidine owing to an enhanced eGFR, which results in enhanced drug clearance. In critically ill children, standard antibiotic dosing may not achieve optimal exposure due to this ARC. Nevertheless, dose optimization for pediatric patients with ARC is scarce due to a lack of PK studies; only one study has previously been reported in children with ARC, aged 2.0–11.8 years (Zhi et al., 2018). To date, the pharmacokinetics of cefathiamidine are lacking in infants with ARC.

Thus, this study intended to establish a population PK model of cefathiamidine suitable for infants with ARC and to determine optimal dosing regimens for these infants.

## Methods

### Study Design

This open-label, single-center PK study of cefathiamidine was performed at Children’s Hospital of Hebei Province affiliated to Hebei Medical University, China. Subjects were included: Infants (≤2 years) with ARC (eGFR ≥ 130 ml/min/1.73 m^2^); these infants received intravenous cefathiamidine as a routine antimicrobial treatment (suspected or confirmed bacterial infections). Subjects were excluded if they had intolerance or allergic reactions to cefathiamidine or were enrolled in other clinical trials. This clinical study of cefathiamidine was approved by the ethics board of hospital.

### Dosing Regimen and Pharmacokinetic Sampling

Cefathiamidine Injection (Xianlisu^®^, Guangzhou Baiyunshan Pharmaceuticals, Guangzhou, China) was administered twice daily as a 30 min intravenous infusion of 100 mg/kg/day. The scavenged sampling approach was utilized to exclusively obtain the residual blood specimens after routine biochemical examination (Zhao and Jacqz-Aigrain, 2015), without additional study-specific blood sampling. The samples were spun down for 5 min at 10,000 rpm, separated and stored frozen at −80 °C. Clinical data and sample information were accurately recorded in a database: age, sex, weight, height, serum creatinine, administration time and sampling time.

### Method of Cefathiamidine Analysis

Concentrations of cefathiamidine were determined by UPLC-MS/MS. The samples were prepared using ceftiofur as internal standard and methanol as deproteination reagent. The separation was achieved using methanol-water as the mobile phase in gradient mode. The m/z in multiple reaction monitoring transitions were 473.5^+^ – 201.3^+^ for cefathiamidine and 524.3^+^ – 241.4^+^ for ceftiofur. The linearity range of cefathiamidine assay based on 50 μl plasma was 30–10,000 ng/ml. The intra- and inter-day coefficients of variation for control samples did not exceed 5% and 15%, respectively. The lower limit of quantification (LLOQ) was 30 ng/ml. The method was validated according to the US FDA guideline (US FDA, 2018) (see Supplementary Material).

### Cefathiamidine Population Pharmacokinetic Modeling

NONMEM V 7.4 software program (Icon Development Solutions, Ellicott City, MD, Unites States) was applied to analyze cefathiamidine PK data. The first-order conditional estimation (FOCE-I) with interaction algorithm was used to assess PK parameters in the model-building.

Inter-individual variability was assessed for the PK parameters by an exponential equation:

$$\theta_{i}=\theta *e^{\eta i}.$$

Here, *θ*_*i*_ is the estimated parameter for the i^th^ subject, *θ* represents the typical population parameter value and *ηi* the interindividual variability which is assumed to be a normal distribution with a mean of zero and variance ω2.

For residual error model, we attempted to evaluate exponential, additive and combined (proportional plus additive) error forms. One- and two-compartment models were initially compared to obtain the appropriate basic PK model. Allometric exponents were explored for weight on clearance (CL) and volume of distribution (V) by fixed (allometric exponents of 0.75 and one for CL and V, respectively) (Holford et al., 2013) and estimated analysis methods. After that, the potential covariates (weight, age, eGFR and sex) on PK parameters were investigated by a stepwise forward selection - backward deletion method (Mandema et al., 1992). The eGFR from serum creatinine was calculated using the Schwartz formula (Schwartz et al., 1987). In the stepwise fashion, the likelihood ratio test was applied to evaluate the influence of covariates on population model parameters. A covariate was considered if a statistically significant (*p* < 0.05, *χ*2 distribution with one degree of freedom) decreasing (reduction > 3.84) objective function value (OFV) for the forward addition step. All statistically significant covariables were incorporated into the full model and then were further evaluated in the backward deletion step. If a covariance was deleted which led to a significant (*p* < 0.01, *χ*2 distribution with one degree of freedom) rise (< 6.635) in OFV, the covariant was eventually excluded from the full model.

The PK model was validated by statistical and graphical approaches. Goodness-of-fit plots, comprising conditional weighted residuals (CWRES) vs time, CWRES vs population prediction (PRED), observed (DV) vs PRED, DV vs individual prediction (IPRED), were used for diagnostics (Hooker et al., 2007). The sampling importance resampling (SIR) analysis with M = 5,000, 2000, 2000, 1,000 samples and m = 1,000, 1,000, 1,000, 500 resamples (4 iterations) was conducted to evaluate the stability and accuracy of the parameter estimates by sir-package in PsN (v5.0.0) software (Dosne et al., 2016; Dosne et al., 2017). RStudio 1.4 using R 3.6.1 was used for graphical output. The convergence of SIR procedure was assessed by the dOFV distribution. The dOFV was the difference between the objective function value of the parameter vector and the OFV of the final parameter estimates. The parameter estimates (median and 95% confidence intervals) from the SIR analysis were contrasted with the parameter values from the original dataset. The normalized prediction distribution error (NPDE) was also applied to evaluate the final PK model (Comets et al., 2008). The original datasets were simulated 1,000 times using parameters from the final PK model. The NPDE results were based on the default graphical summary provided by the NPDE R package (v1.2) (Comets et al., 2008): 1) QQ-plot of the NPDE; 2) histogram of the NPDE. The NPDE was assumed to follow the N (0, 1) distribution.

### PTA-Based Optimization of Dosing Regimen

The percentage of time that free drug concentration is above MIC for the dosing interval (fT_MIC_) is important for the therapeutic efficacy of β-lactams (Hoog et al., 2005). The maximum antibacterial effect of β-lactams was assumed to be attained when the free fraction of drug exceeds the MIC for 60%–70% of dosing interval (Craig, 1998; Drusano, 2004). The 70% fT_MIC_ target was used as a conservative pharmacodynamic endpoint for infants.

Considering the balance between maximum efficacy, minimum toxicity and reduction of resistance, the following pharmacokinetic-pharmacodynamic target was chosen: 70% of patients attained the target of 70% fT_MIC_ (Cohen-Wolkowiez et al., 2012; Zhi et al., 2018; Shi et al., 2020). A fixed unbound fraction of 77% was used to calculate fT_MIC_ in this study (Tze-ying et al., 1979). Cefathiamidine is used to treat severe and often life-threatening infections in pediatric patients caused by *Streptococcus pneumoniae* (MIC_90_ 0.25 mg/L); *Streptococcus pyogenes* (MIC_90_ 0.5 mg/L); *H. influenza, Moraxella catarrhalis* and *Enterococcus* (MIC_90_ 2 mg/L); MSSA and MSSE (MIC_90_ 8 mg/L) for susceptible isolates (Guangzhou Baiyunshan Pharmaceuticals, 2015). Monte Carlo simulations (*n* = 1,000) were performed for various dosing regimens in infants by utilizing the original datasets to calculate the target attainment rate for the following MICs: 0.25, 0.5, 2, and 8 mg/L. The dose of cefathiamidine was simulated on an mg/kg basis. Target attainments rates were calculated for simulated doses to explore the PTA - based dosage regimen in infants with ARC.

## Results

### Study Population

In total, 20 infants with ARC who underwent cefathiamidine treatment were recruited in this PK study. All infants received cefathiamidine as an intravenous infusion at an administered dose of 100 mg/kg/day q12 h. The median (range) eGFR of infants was 197 (132–413) ml/min/1.73 m^2^. Weight and age were all normally distributed in this study (*p* = 0.20 and *p* = 0.08, respectively, Kolmogorov-Smirnov test). The mean (SD) values of age and weight in the infants were 1.20 (0.43) (range 0.35–1.86) years and 10.33 (1.57) (range 8.0–12.5) kg, respectively. The patient characteristics are presented in Table 1.

**TABLE 1:** Baseline characteristics in 20 infants.

Characteristics	Number	Mean (SD)	Median (Range)
Patients	20		
Male/female	10/ 10		
Race	Chinese		
Age (years)		1.20 (0.43)	1.25 (0.35–1.86)
Current weight (kg)		10.33 (1.57)	10.25 (8.00–13.00)
Scr (µmol/L)		18 (6)	20 (10–26)
eGFR (mL/min/1.73 m^2^)		230 (86)	197 (132–413)
Dose (mg/dose)		533 (167)	500 (400–1,000)
Dose (mg/kg/dose)		52 (16)	50 (40–100)
Hematologic disease			
Immune thrombocytopenia	6		
Leukemia	3		
Anemia	3		
Infectious mononucleosis syndrome	2		
Agranulocytosis	2		
Other	4		

Notes: Scr: Serum creatinine concentration; eGFR: Estimated glomerular filtration rate.

### Model Building

For the population modeling, 36 cefathiamidine blood samples, with concentrations ranging from 0.15 to 222.00 μg/ml, were available. The concentrations of all samples were above the LLOQ. The concentration on log scale vs time profile of cefathiamidine is presented in Supplementary Figure S1.

The PK data of cefathiamidine were adequately illustrated by a 1-compartment model with first-order elimination. The model parameters were estimated regarding CL and V. For cefathiamidine, inter-individual variability (IIV) was exponentially modeled and then estimated for V and CL. An exponential model best described residual variability.

### Covariate Analysis

The weight with allometric scaling approach was incorporated into the basic model (fixed allometric exponents of 0.75 and 1 for CL and V, respectively), with a significant decrease in the OFV of 7.37 points. Age was the most critical covariate on CL, along with a further OFV drop of 13.61 points and IIV drop of 15%. The *η*- shrinkages of the final PK model were 15.1 and 28.5% for CL and V, respectively. Table 2 presents detailed parameter estimates for the final PK model.

**TABLE 2:** Population PK parameters of cefathiamidine and SIR results.

Parameters	Full dataset		SIR	
	Final estimate	RSE (%)	Median (RSE%)	95% CIs
CL (L/h)				
CL = *θ*1×(CW/10.25)^0.75^×F_age_				
*θ*1	2.20	8.30	2.21 (8.2)	1.87–2.58
V (L)				
V = *θ*2× (CW/10.25)				
*θ*2	3.36	8.2	3.35 (7.9)	2.89–3.96
F_age_= (AGE/1.25)^*θ*3^				
*θ*3	0.662	21.6	0.651 (22.5)	0.358–0.931
Inter-individual variability (shr) (%)				
CL	25.6 (15.1)	14.3	27.2 (18.2)	18.0–34.5
V	22.4 (28.5)	28.6	23.1 (38.4)	5.00–38.2
Residual variability (shr) (%)				
ERR (1)	22.6 (35.4)	22.2	23.3 (27.7)	9.81–34.7

Notes: CL: clearance; V: volume of distribution; CW: current weight in kilogram; Fage: age factor; AGE: age in years shr: shrinkage in %. In our population, 10.25 kg and 1.25 years are the median current weight and age values on the day of first sampling, respectively.

The median (range) of weight-normalized CL and V were 0.22 (0.09–0.29) L/h/kg and 0.34 (0.24–0.41) L/kg, respectively. Cefathiamidine CL (L/h) increased allometrically with weight (kg) in infants. Cefathiamidine weight-normalized CL (L/h/kg) also increased with age (years) (Supplementary Figure S2). The area under the curve from time 0 to 24 h (AUC_0-24_) for the prescribed dose ranged from 296 to 1,152 mg*h/L at steady-state.

### Model Evaluation

**FIGURE 1:**
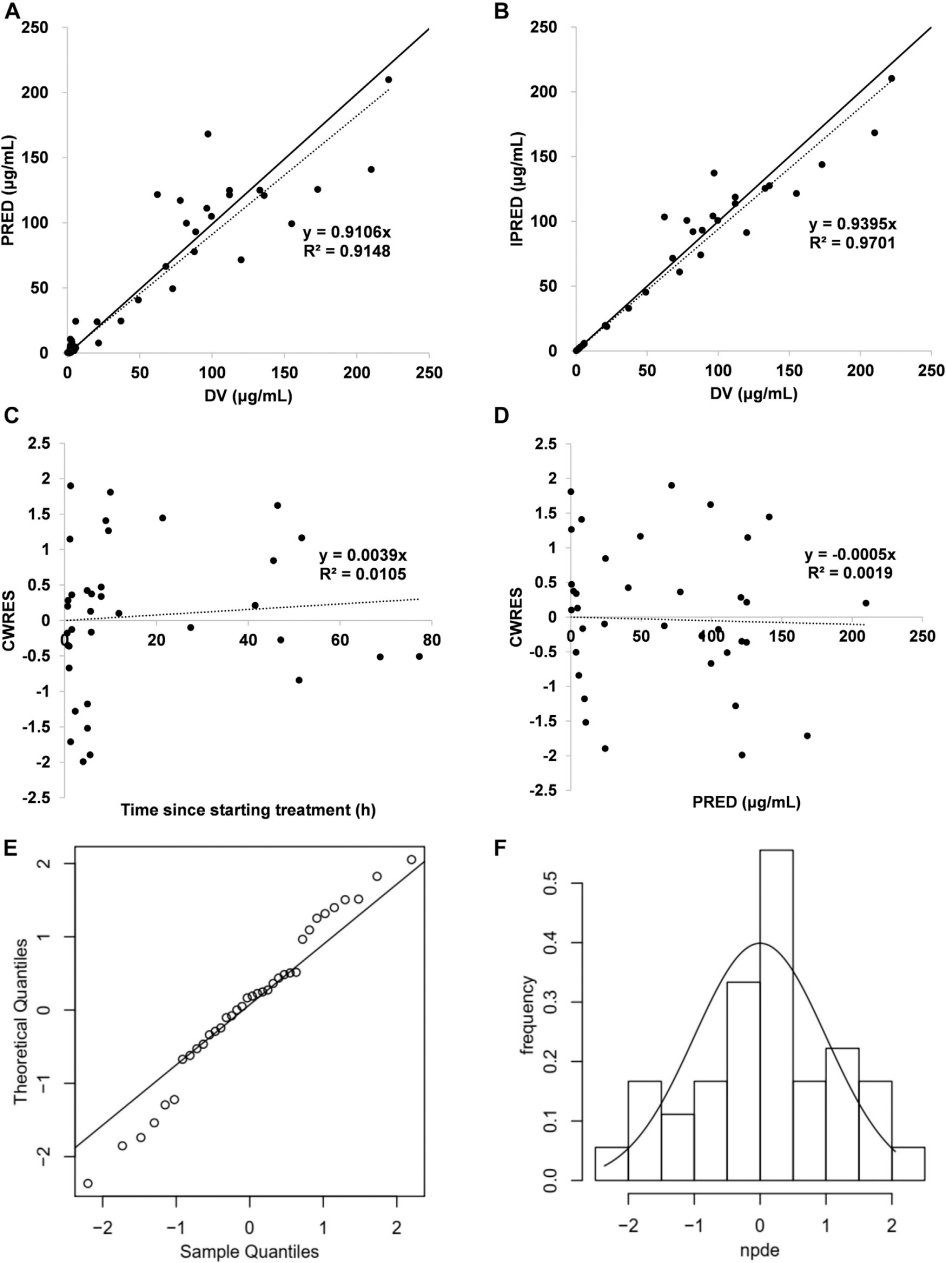
Model evaluation for cefathiamidine **(A)** PRED vs DV **(B)** IPRED vs DV **(C)** CWRES vs time **(D)** CWRES vs PRED **(E)** NPDE QQ-plot vs the theoretical N (0,1) distribution **(F)** NPDE distribution histogram with the density of the standard Gaussian distribution overlaid. In the plot, the solid line is the identity line and the dotted line is the trend line.

An acceptable goodness-of-fit of the final model was shown in Figures 1A–D. In the plots of PRED vs DV and IPRED vs DV, a symmetric distribution of points was observed around the identity line. The plots of CWRES vs PRED and CWRES vs time were randomly distributed around CWRES = 0 within the residuals range from -2 to 2. No bias was observed in goodness-of-fit plots. The dOFV plot showed that the proposal distribution was above the reference Chi square and that the dOFV distributions of the resamples of last two iterations were overlaid. The dOFV plot was shown in Supplementary Figure S5. The final parameter estimates were close to the median SIR analysis values and lay within 95% confidence intervals (CIs) obtained from the SIR analysis, demonstrating that the developed model was robust (Table 2). The NPDE distribution and histogram comply well with the distribution and density of theory N (0, 1), indicating the model fits well with the individual data (Figures 1E,F). The variance and mean of NPDE were 1.14 and 0.09, respectively. The value of Fisher variance test, Wilcoxon signed rank test, Shapiro-Wilks test of normality and global-adjusted *p*-value is 0.533, 0.571, 0.614 and 1, respectively.

### PTA-Based Dosing Regimen Optimization and Evaluation

**FIGURE 2:**
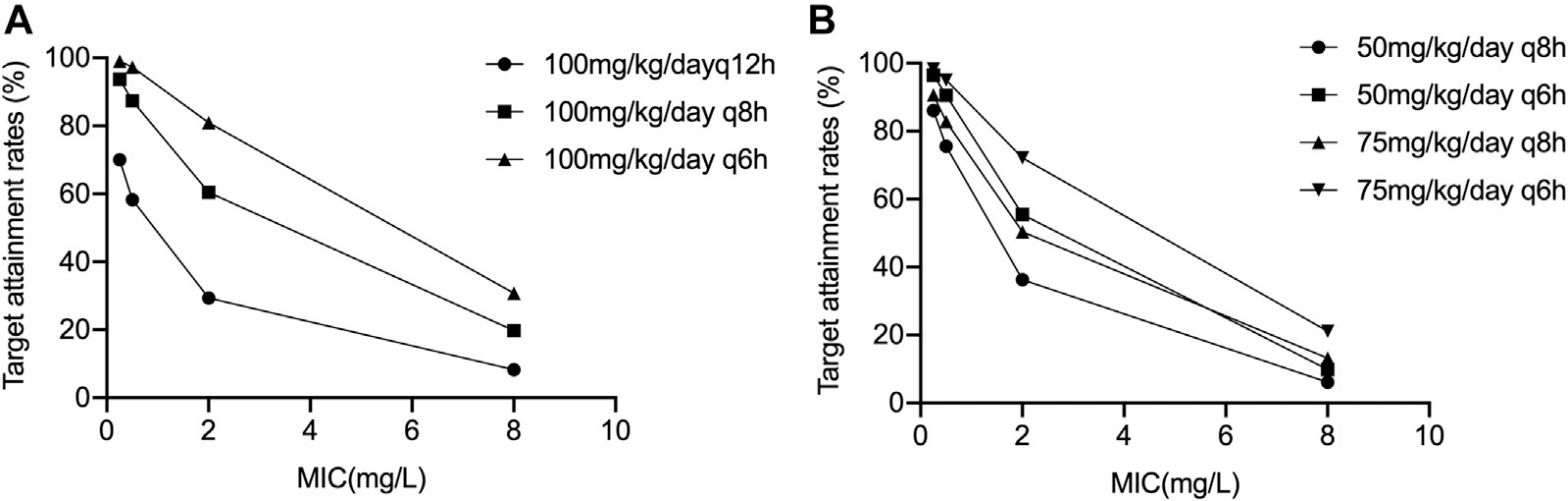
Results of the PTA-based dosing simulations **(A)** 100 mg/kg/day q6 h, q8 h, and q12 h; **(B)** 50 mg/kg/day q6 h, q8 h, and 75 mg/kg/day q6 h, q8 h.

Results of the PTA-based dosing simulations are showed in Figure 2. For the prescribed dose of 100 mg/kg/day q12 h, the target (70% fTMIC) was achieved in 70.1, 58.3, 29.4 and 8.3% of infants for bacteria with a MIC of 0.25, 0.5, 2 and 8 mg/L, respectively. If the dosing interval was shortened to 8 h, the doses of 50 mg/kg/day q8 h resulted in 75.5% (MIC 0.5 mg/L) and 36.3% (MIC 2 mg/L) infants to achieving the target, respectively. If the dosing interval was shortened to 6 h, the doses of 75 mg/kg/day q6 h resulted in 72.1% (MIC 2 mg/L) of infants achieving the target. Nevertheless, the dose of 100 mg/kg/day q6 h resulted in only 30.8% of infants achieving the target for bacteria with a MIC of 8 mg/L, indicating the need for higher dosing or different antibiotics.

## Discussion

For the first time a PK model of intravenous cefathiamidine was established in infants with ARC that was also used for dose optimization. A one-compartment model best fitted the PK data obtained from 20 ARC infants. The median CL and V of cefathiamidine in infants aged 0.35–1.86 years were 0.22 L/h/kg and 0.34 L/kg, respectively. The final model was verified by graphics and statistical methods, which showed that the model had a good prediction performance and stability.

Cefathiamidine is primarily eliminated by the renal pathway as the parent compound, and GFR as an indicator of renal function may influence cefathiamidine disposition. Nevertheless, the covariate screening analysis showed that eGFR had no significant influence on cefathiamidine clearance. Only weight with allometric scaling and age were identified as significant covariates. This can be ascribed to a limited range of eGFR (132–413 ml/min/1.73 m^2^). The plot between cefathiamidine CL and eGFR shows no trend (Supplementary Figure S3).

It is noteworthy that the infants with ARC (eGFR, range 132–413 ml/min/1.73 m^2^) were included in the current study. The primary mechanisms underlying ARC are likely to be a result of the systemic inflammatory response, hyperdynamic cardiovascular state, fluid volume loading characterized by increases in cardiac output and renal blood flow (Udy et al., 2010; Cook and Hatton-Kolpek, 2019). ARC is associated with enhanced drug elimination (Zhou et al., 2020) and, as a consequence, underexposure of patients to renally excreted medications. We summarized eight pharmacokinetic models of renally eliminated drugs in pediatric patients with ARC, as shown in Supplementary Tables S1, S2. Importantly, clinicians need to be aware of the risk of conventional dosing in patients with ARC because these patients have elevated significant higher CL than the general population without ARC for renally cleared drugs (Udy et al., 2015; Lv et al., 2020). Age and ARC had effects on the pharmacokinetic parameters of renally excreted drugs (Avedissian et al., 2017; Chen et al., 2018; Béranger et al., 2019). The estimated CL (0.09–0.29 L/h/kg) in this study is different from the CL value (0.05–0.43 L/h/kg) in children reported previously. (Zhi et al., 2018). This difference is likely due to the effect of the age groups of infants and children on CL. In this study, we analyzed the CL difference between infants ≤1 year old and those 1–2 years old, with the *p* value set a priori at 0.05. The mean (SD) of CL values were 0.11 (0.032) L/h/kg and 0.23 (0.034) L/h/kg for infants ≤1 and 1–2 years old, respectively, and the difference between two age groups was statistically significant (independent samples t test, t = − 6.424, *p* ＜ 0.05).

Simulations showed that the current dosage of cefathiamidine (100 mg/kg/day q12 h) would lead to a high risk of underdosing in infants with ARC for bacteria with a MIC ≥ 0.5 mg/L. To improve the proportion of patients reaching the pharmacodynamic target, increasing the dose and/or dosing frequency have been selected (Shi et al., 2018; Li et al., 2019). As the safety of high doses and toxicity threshold has not been evaluated, increasing the dosing frequency has been primarily considered to avoid possible cefathiamidine related toxicity. The optimal dosing regimens of 50 mg/kg/day q8 h and 75 mg/kg/day q6 h was required to treat bacteria with a MIC 0.5 and 2 mg/L, respectively. When the MIC was 8 mg/L, the therapeutic target is difficult to achieve, and different antibiotics should be taken into consideration in clinical treatment. In this study, the cumulative fraction of response (CFR) was not calculated to estimate the overall response of microorganisms to cefathiamidine, due to the lack of study on MIC distributions for strains. The MIC distribution for cefathiamidine with respect to strains should be studied in the future. In respiratory infection, the most common microorganisms were *H. influenza* (33.90%), *Streptococcus pneumoniae* (33.55%), *Moraxella catarrhalis* (19.20%) and *Staphylococcus aureus* (3.64%) from 15047 children (Wang et al., 2019). About 90% of the common pathogens had MIC90 ≤ 2 mg/L. The dose of 75 mg/kg/day q6 h was recommended for respiratory infection.

Our study has several limitations. First, the PK model of cefathiamidine was only validated internally due to a limited number of patients. Second, the unbound concentration of cefathiamidine was not measured due to the limited sample volume, and the total concentration was analyzed Given that cefathiamidine has a low protein binding ratio of 23%, albumin collection was not included in the design of the PK study. We eventually adopted a fixed unbound fraction of 77% in dosing simulation. Third, the GFR was estimated based on serum creatinine in our study, because timed urine collection is difficult in infants who are not toilet trained or have bladder dyssynergia. Finally, PK study of cefathiamidine was not available in the non-ARC pediatric population, and further studies should be performed in larger pediatric patients with and without ARC. The clinical application of dose optimization based on PK modeling should be further evaluated in the clinical setting.

## Conclusion

The population PK model of cefathiamidine was developed in infants with ARC. Weight with allometric scaling and age have been shown to have significant effects on cefathiamidine pharmacokinetics. The prescribed dose (100 mg/kg/day q12 h) could cover bacteria with a MIC ≤ 0.25 mg/L. Based on this developed PK model, 50 mg/kg/day q8h and 75 mg/kg/day q6h were adopted for bacteria with MIC 0.5 and 2 mg/L to achieve the pharmacodynamic target, respectively. As the relationship between high dose and safety remains to be revealed, other antibiotics should be considered for bacteria with a MIC of 8 mg/L and higher.

## Data Availability

The raw data supporting the conclusions of this article will be made available by the authors, without undue reservation.
