# Supplementary material for: Population Pharmacokinetic Study of Cefathiamidine in Infants With Augmented Renal Clearance
Source: Front Pharmacol. 2021 Mar 15;12:630047. doi: 10.3389/fphar.2021.630047 (PMC8005605; doi:10.3389/fphar.2021.630047)
Supplement: Supplementary file 1 [file datasheet1.docx]

**Supplementary material**

**Analysis of Cefathiamidine**

The validation of cefathiamidine quantitative method conformed to US Food and Drug Administration guidelines (US FDA, 2018), including selectivity, linearity, intra- and inter-day precision and accuracy, LLOQ, stability, recovery, matrix effect.

Selectivity was investigated by assessing blank plasma from six individuals. No significant interference was observed at the elution time of two analytes. The retention time for IS and cefathiamidine were 0.54 min and 0.75 min with a total running time of 1.8 min. Calibration curve of cefathiamidine (30 to 10,000 ng/mL) was linear, with R^2^＝0.99. The accuracy and CV of LLOQ were 102.2.1% and 11.9%, which were acceptable. The CV and accuracy were performed on low, medium and high level quality control (QC) samples (n =6 for each). The Intra- and inter-day accuracy of samples were in the range 85 - 115%. The intra- and inter-day CV of QC samples did not exceed 5% and 15%, respectively. Stability studies were performed at three QC levels according to the reported method ([Zhuang et al., 2019](#_ENREF_1)). The stability results indicated that the analyte was stable under test conditions. The recoveries on six replicates of three QC concentration levels ranged from 91.69 to 106.56%. Matrix effects of cefathiamidine and IS were calculated by the ratio of the peak areas in the presence of plasma to the peak areas in the absence of plasma. The CV of matrix effects between the three QC concentration levels were all below 15%.

**Table Legends**

**Supplementary Table S1 and S2**: Pharmacokinetic models of renally eliminated drugs in pediatric patients with ARC.

**Figure** **Legends**

**Supplementary Figure S1**: Cefathiamidine concentration on log scale vs time**.**

**Supplementary Figure S2**: Cefathiamidine weight-normalized CL vs age**.**

**Supplementary** **Figure S3:** Cefathiamidine weight-normalized CL vs eGFR.

**Supplementary Figure S4:** Model evaluation for cefathiamidine. **(A)** Log_10_(PRED) vs Log_10_(DV). **(B)** Log_10_(IPRED) vs Log_10_(DV). **(C)** CWRES vs time. **(D)** CWRES vs PRED. In the plot, the black dotted line is loess smoother line and the gray dotted line is identity line.

**Supplementary Figure S5:** The dOFV plot showed that the proposal distribution was above the reference chi-square distribution and that SIR had converged (SIR distribution of iterations 3 and 4 overlaid).

| Reference | Lv et al., 2020 | Avedissian et al., 2017 | Zhao et al., 2014 | Wang et al., 2020a |
| --- | --- | --- | --- | --- |
| Drugs | vancomycin | vancomycin | vancomycin | vancomycin |
| Patients | ARC children (age range 2.24-17.87 years) | ARC and non-ARC infants and children (age range 1-12 years) | Infants and children (age range 0.3-17.7 years) | Infants and children (age range 0.4-17.1 years) |
| Creatinine Clearance  or  Serum Creatinine | Creatinine Clearance  Range 133.07-1283.6  (ml/min/1.73m2) | Creatinine Clearance  median (IQR)  ARC 141.3(132.7-1489.9)  Non-ARC 91.7(74.8-106.6)  (ml/min/1.73m2) | Creatinine Clearance  Range 48.7-457  (ml/min/1.73m2) | Creatinine Clearance  Range 58.9-274.3  (ml/min/1.73m2) |
| No. of patients | 53 | Overall n=250  ARC n=29  Non-ARC n=221 | 70 | 92 |
| No. of samples | 106 | 658 | 98 | 144 |
| One- or two-compartment  model | One | One | One | One |
| Significant covariate  on CL | Weight | Weight and SCr | Weight and CLcr | Weight and eGFR |
| CL formula | 6.32×(WT/70)^0.75^ × e^0.0467^ | 0.188×(WT)× e^-1.13^×(SCr-0.40) | 4.37×(WT/20.2)^0.677^×  (CLcr/191)^1.03^ | 4.18×(eGFR/145)^0.741^×(WT/25)^K^,  K= WT^-0.856^/(WT^-0.856^+6.53^-0.856^) |
| Significant covariate on V | Weight | Weight | Weight | _ |
| V formula | 39.6×(WT/70) | 0.624 ×WT | 119×(WT/20.2)^0.838^ | 22.3 |
| Model validation | Internal | Internal | Internal and external | Internal |
| MPE  (5th to 95th)  MAPE | MPE 404.1%  (-91.4% -10276.6%)  MAPE 493.9% | MPE 2518.8%  (-46.3% - 51788.5%)  MAPE 2542.5% | MPE 1036.1%  (-71.7% - 27635.0%)  MAPE 1063.5% | MPE 1398.6%  (-84.4% - 32713.3%)  MAPE 1063.5% |

**Table S1**: Pharmacokinetic models of renally eliminated drugs in pediatric patients with ARC.

**Table S2**: Pharmacokinetic models of renally eliminated drugs in pediatric patients with ARC.

| Reference | Li et al., 2019 | Wang et al., 2020b | Zhao et al., 2015 | Wang et al., 2020c |
| --- | --- | --- | --- | --- |
| Drugs | Linezolid | ceftriaxone | teicoplanin | meropenem |
| Patients | Infants and children (age range 0.03-11.9 years) | Infants and children (age range 0.1-1.99 years) | Infants and children (age range 0.5 – 16.9 years) | Infants and children (age range 0.101– 14.4) |
| Creatinine Clearance  or  Serum Creatinine | Creatinine Clearance  Range 45.1-338.1  mL/min/1.73 m 2 | Serum Creatinine  3.0-39 µmol/L | Creatinine Clearance  Range 48.6- 464.1  mL/min | Creatinine Clearance  Range 57.9 – 279  ml/min/1.73m2 |
| No. of patients | 112 | 66 | 85 | 57 |
| No. of samples | 135 | 169 | 143 | 135 |
| One- or two-compartment  model | One | One | Two | Two |
| Significant covariate  on CL | Weight and eGFR | Weight and age | Weight and CLcr | Weight and CLcr |
| CL formula | 1.31×(lnWT/2.40)^0.83^×  (lneGFR/4.89)^0.6^ | 0.27×(WT/9.85)^0.44^×  (age/7)^0.21^ | CL=0.491×(WT/27.1)^0.75^×  (CLcr /179)^0.606^  Q=0.341×(WT/27.1)^0.75^ | CL=4.79×(WT/11)^0.365^×  (CLcr /155.6)^0.317^  Q=0.216 |
| Significant covariate on V | Weight | _ | Weight | Weight |
| V formula | 4.24×(ln WT/2.40)^0.86^ | 1.72 | V1=4.18×(WT/27.1)  V2=1.3×(WT/27.1) | V1=4.18×(WT/11)  V2=1.67 |
| Model validation | Internal | Internal | Internal | Internal |
| MPE  (5th to 95th)  MAPE | MPE 47.4%  (-5.5% - 502.2%)  MAPE 49.4% | MPE 2977.7%  (0.152% - 64159.4%)  MAPE 2978.5% | MPE 1940.4%  (-17.9% - 39983.0%)  MAPE 1946.8% | MPE 20.5%  (-68.7% - 26.5%)  MAPE 23.7% |


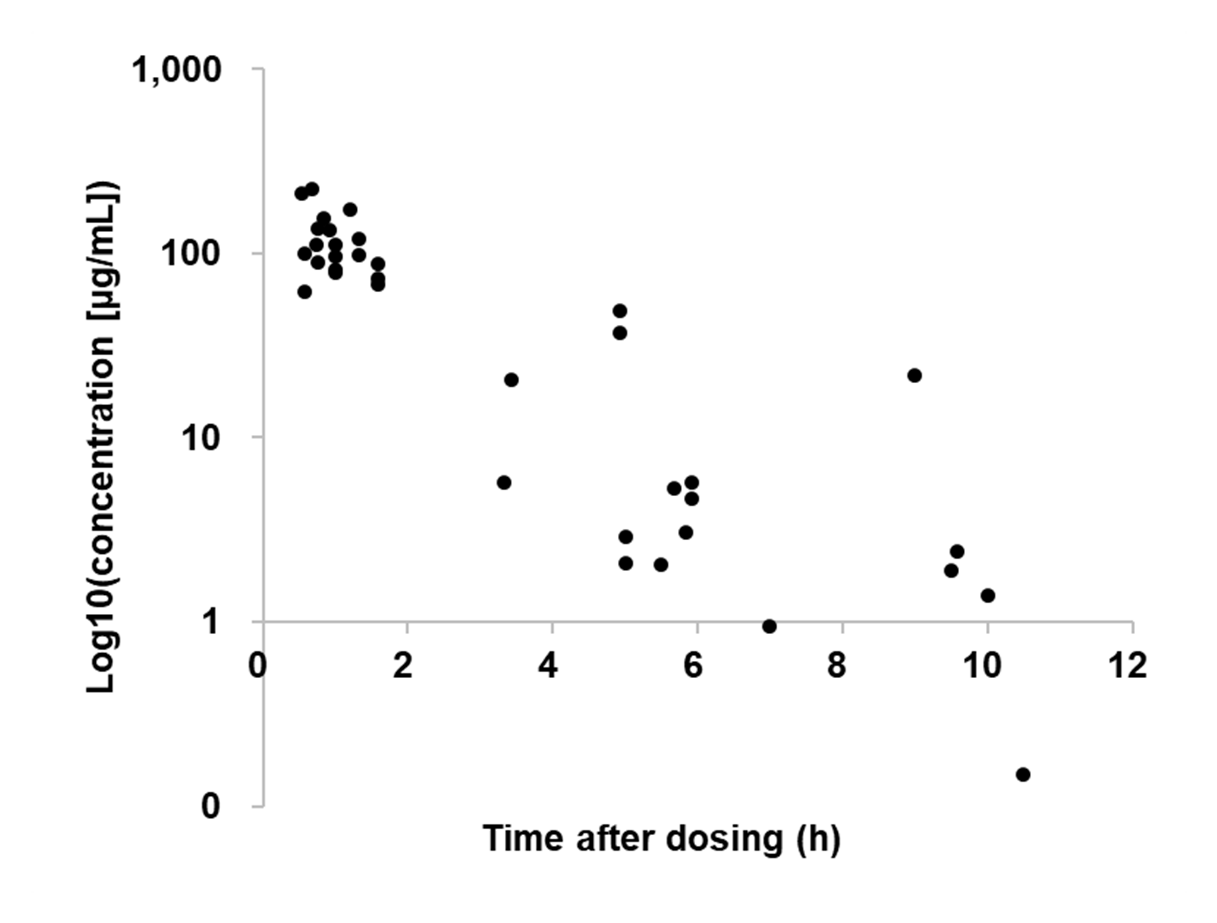


**Figure S1**: Cefathiamidine concentration on log scale vs time.


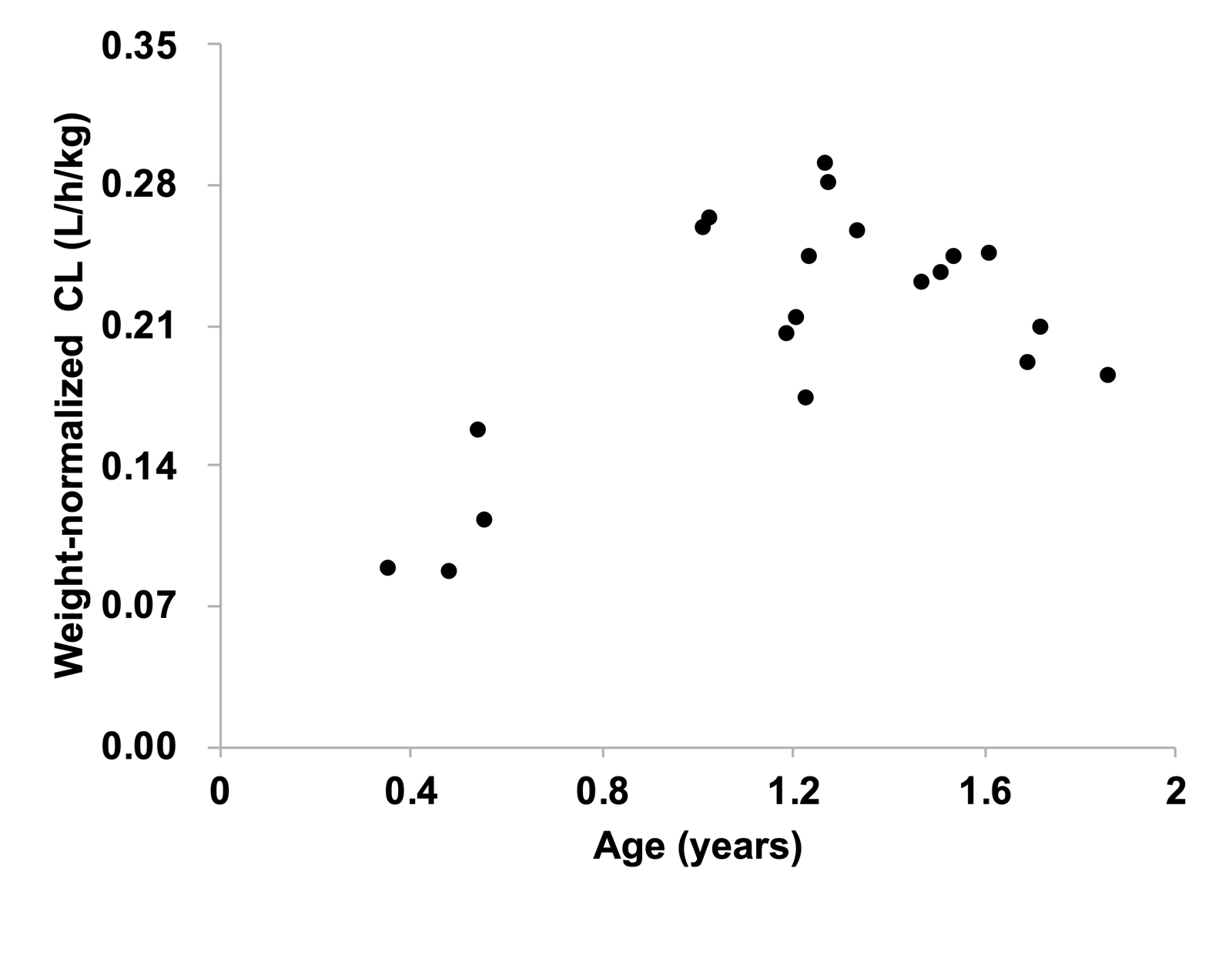


**Figure S2**: Cefathiamidine weight-normalized CL vs age.





**Figure S3**: Cefathiamidine weight-normalized CL vs eGFR.


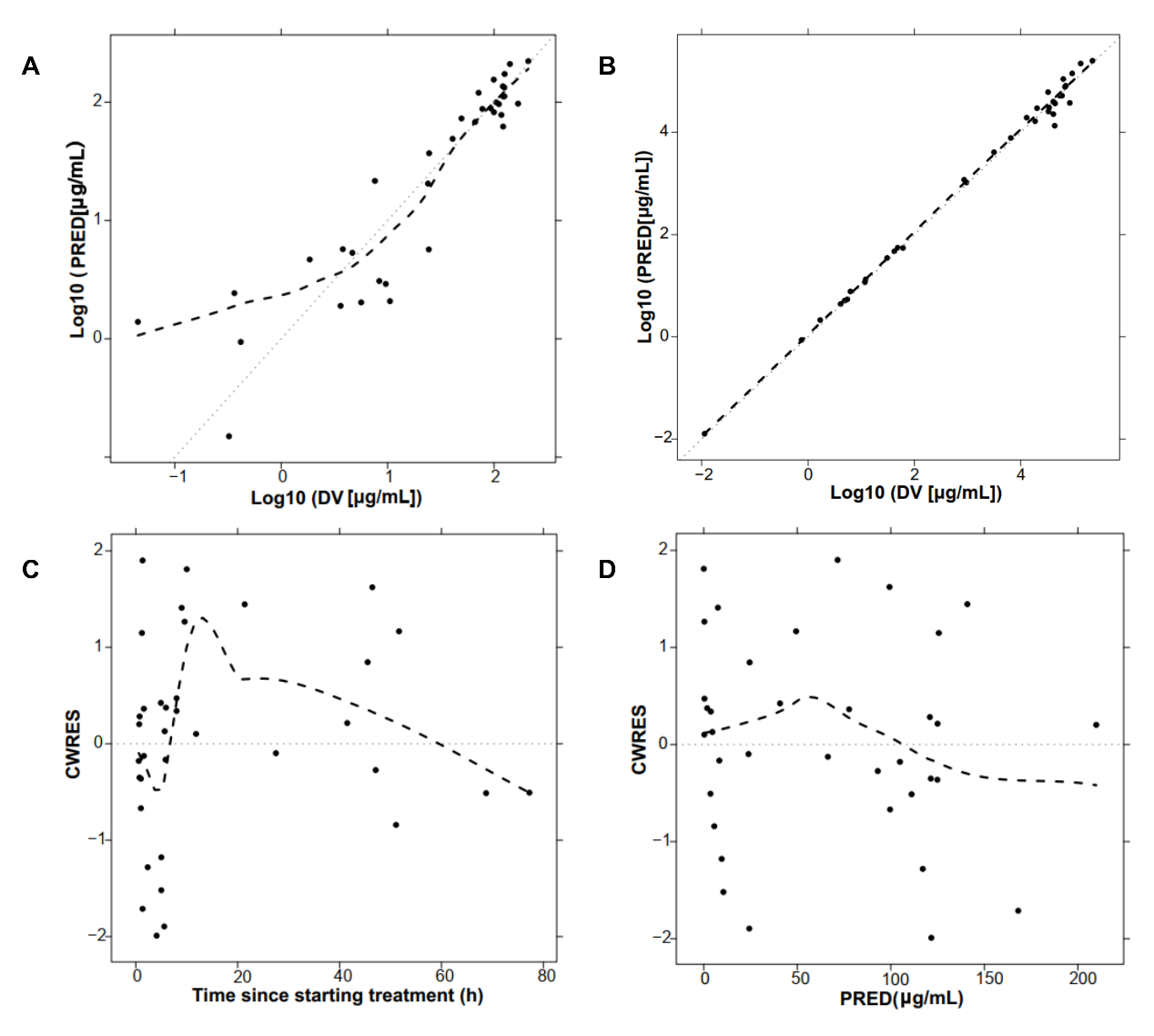


**Figure S4**: Model evaluation for cefathiamidine. **(A)** Log_10_(PRED) vs Log_10_(DV). **(B)** Log_10_(IPRED) vs Log_10_(DV). **(C)** CWRES vs time. **(D)** CWRES vs PRED. In the plot, the black dotted line is loess smoother line and the gray dotted line is identity line.


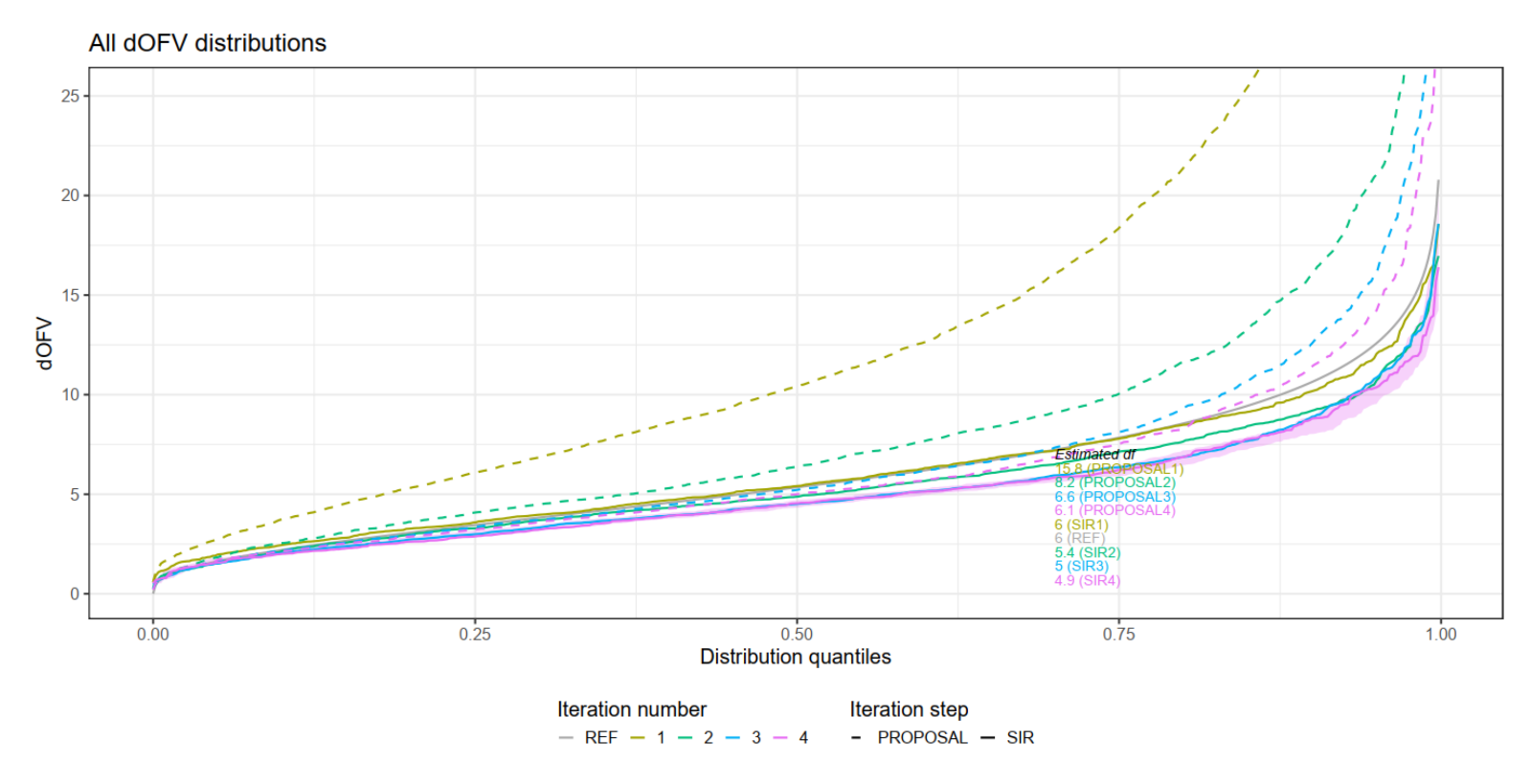


**Figure S5:** The dOFV plot showed that the proposal distribution was above the reference chi-square distribution and that SIR had converged (SIR distribution of iterations 3 and 4 overlaid).

**References**

Avedissian, S.N., Bradley, E., Zhang, D., Bradley, J.S., Nazer, L.H., Tran, T.M., et al. (2017). Augmented Renal Clearance Using Population-Based Pharmacokinetic Modeling in Critically Ill Pediatric Patients. *Pediatric Critical Care Medicine* 18(9)**,** e388-e394. doi: 10.1097/PCC.0000000000001228.

Li, S.-C., Ye, Q., Xu, H., Zhang, L., and Wang, Y. (2019). Population pharmacokinetics and dosing optimization of linezolid in pediatric patients. *Antimicrob Agents Chemother* 63(4)**,** e02387-02318. doi: 10.1128/AAC.02387-18.

Lv, C.-L., Lu, J.-J., Chen, M., Zhang, R., Li, Q.-C., Chen, Y.-Y., et al. (2020). Vancomycin population pharmacokinetics and dosing recommendations in haematologic malignancy with augmented renal clearance children. *Journal of Clinical Pharmacy and Therapeutics* 45(6)**,** 1278-1287. doi: 10.1111/jcpt.13206.

US FDA. (2018). Guidance for industry: bioanalytical method validation. https://www.fda.gov/downloads/Drugs/Guidance/ucm070107.pdf

Wang, H., Huang, L., Wang, J., Ni, Y., Zhu, Z., Gao, P., et al. (2020a). Population Pharmacokinetic Study of Vancomycin in Chinese Pediatric Patients with Hematological Malignancies. *Pharmacotherapy* 40(12)**,** 1201-1209. doi: 10.1002/phar.2473.

Wang, Y.-K., Wu, Y.-E., Li, X., Tian, L.-Y., Khan, M.W., Tang, B.-H., et al. (2020b). Optimal dosing of ceftriaxone in infants based on a developmental population pharmacokinetic-pharmacodynamic analysis. *Antimicrob Agents Chemother* 64(11)**,** e01412-01420. doi: 10.1128/AAC.01412-20.

Wang, Z.-M., Chen, X.-Y., Bi, J., Wang, M.-Y., Xu, B.-P., Tang, B.-H., et al. (2020c). Reappraisal of the optimal dose of meropenem in critically ill infants and children: a developmental pharmacokinetic-pharmacodynamic analysis. *Antimicrob Agents Chemother* 64(8)**,** e00760-00720. doi: 10.1128/AAC.00760-20.

Zhao, W., Zhang, D., Fakhoury, M., Fahd, M., Duquesne, F., Storme, T., et al. (2014). Population pharmacokinetics and dosing optimization of vancomycin in children with malignant hematological disease. *Antimicrob Agents Chemother* 58(6)**,** 3191-3199. doi: 10.1128/AAC.02564-13.

Zhao, W., Zhang, D., Storme, T., Baruchel, A., Declèves, X., and Jacqz-Aigrain, E. (2015). Population pharmacokinetics and dosing optimization of teicoplanin in children with malignant haematological disease. *British journal of clinical pharmacology* 80(5)**,** 1197-1207. doi: 10.1111/bcp.12710.

Zhuang, Q., Pan, R., Liu, X., Xu, W., Wang, H., Zhang, X., et al. (2019). A validated ultra-HPLC-MS/MS method for determination of honokiol in human plasma and its application to a clinical pharmacokinetic study. *Journal of Chromatography B* 11(11)**,** 1085-1098. doi: 10.4155/bio-2019-0030.
